# Exploring functional conservation *in silico*: a new machine learning approach to RNA-editing

**DOI:** 10.1093/bib/bbae332

**Published:** 2024-07-09

**Authors:** Michał Zawisza-Álvarez, Jesús Peñuela-Melero, Esteban Vegas, Ferran Reverter, Jordi Garcia-Fernàndez, Carlos Herrera-Úbeda

**Affiliations:** Departament de Genètica, Microbiologia i Estadística, Facultat de Biologia, Universitat de Barcelona, Av. Digonal 643, 08028 Barcelona, Spain; Institut de Biomedicina (IBUB), Universitat de Barcelona, Av. Diagonal 643, 08028 Barcelona, Spain; Departament de Genètica, Microbiologia i Estadística, Facultat de Biologia, Universitat de Barcelona, Av. Digonal 643, 08028 Barcelona, Spain; Departament de Genètica, Microbiologia i Estadística, Facultat de Biologia, Universitat de Barcelona, Av. Digonal 643, 08028 Barcelona, Spain; Centro de Investigación Biomédica en Red de Fragilidad y Envejecimiento Saludable (CIBERFES), Instituto de Salud Carlos III, Calle Sinesio Delgado 4, 28029 Madrid, Spain; Departament de Genètica, Microbiologia i Estadística, Facultat de Biologia, Universitat de Barcelona, Av. Digonal 643, 08028 Barcelona, Spain; Departament de Genètica, Microbiologia i Estadística, Facultat de Biologia, Universitat de Barcelona, Av. Digonal 643, 08028 Barcelona, Spain; Institut de Biomedicina (IBUB), Universitat de Barcelona, Av. Diagonal 643, 08028 Barcelona, Spain; Departament de Genètica, Microbiologia i Estadística, Facultat de Biologia, Universitat de Barcelona, Av. Digonal 643, 08028 Barcelona, Spain; Institut de Biomedicina (IBUB), Universitat de Barcelona, Av. Diagonal 643, 08028 Barcelona, Spain

**Keywords:** machine learning, deep learning, RNA modification, A-to-I editing, evolution

## Abstract

Around 50 years ago, molecular biology opened the path to understand changes in forms, adaptations, complexity, or the basis of human diseases through myriads of reports on gene birth, gene duplication, gene expression regulation, and splicing regulation, among other relevant mechanisms behind gene function. Here, with the advent of big data and artificial intelligence (AI), we focus on an elusive and intriguing mechanism of gene function regulation, RNA editing, in which a single nucleotide from an RNA molecule is changed, with a remarkable impact in the increase of the complexity of the transcriptome and proteome. We present a new generation approach to assess the functional conservation of the RNA-editing targeting mechanism using two AI learning algorithms, random forest (RF) and bidirectional long short-term memory (biLSTM) neural networks with an attention layer. These algorithms, combined with RNA-editing data coming from databases and variant calling from same-individual RNA and DNA-seq experiments from different species, allowed us to predict RNA-editing events using both primary sequence and secondary structure. Then, we devised a method for assessing conservation or divergence in the molecular mechanisms of editing completely *in silico*: the cross-testing analysis. This novel method not only helps to understand the conservation of the editing mechanism through evolution but could set the basis for achieving a better understanding of the adenosine-targeting mechanism in other fields.

## Graphical Abstract

**Graphical Abstract:**
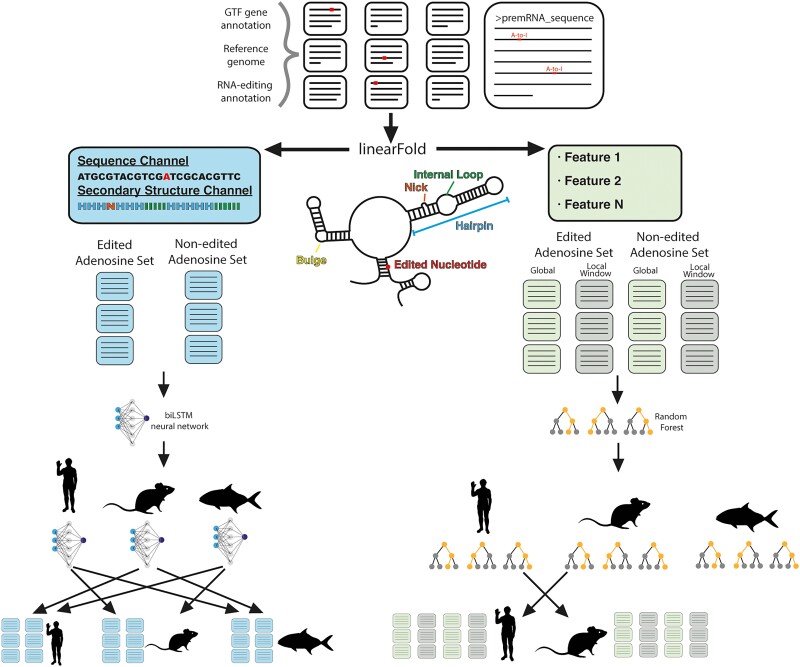

## Introduction

Gene regulation is without a doubt the Rosetta Stone of genetics in the 21st century. Among the different posttranscriptional modifications behind gene regulation, RNA editing has received less attention until a few years ago. This is especially true when compared with others as profoundly studied as alternative splicing [1–5]. In a common RNA-editing event, a single nucleotide from an RNA molecule undergoes a chemical change, turning into a different nucleotide, usually before the RNA molecule undergoes any kind of splicing [6, 7]. This process is present in all eukaryotic organisms [8–10], being the Adenine-to-Inosine (A-to-I) editing mediated by proteins of the ADAR (Adenosine Deaminase Acting on RNA) family, the most common in metazoans [11–13]. The ADAR family includes three paralog groups in vertebrates: ADAR, ADARB1, and ADARB2. ADARB1 and ADARB2 are the result of the two whole genome duplications (WGD) that took place at the origin of vertebrates, with the basal-branching cephalochordate *Branchiostoma lanceolatum* presenting only two of the three commonly found paralogs [14]. Interestingly, out of the expected up to four vertebrate paralogs for each amphioxus gene, only one duplication event was conserved in the vertebrate lineage, with one of the two paralogs, ADARB2, being enzymatically inactive and acting just as a binding competitor [15, 16].

The ADAR-mediated A-to-I modification, although apparently small, with just one nucleotide change, can have various translational consequences. If the editing event takes place inside the coding sequence (CDS), it can have an impact on the final protein as the new inosine will behave like a guanine in any base-pairing process, such as translation [17]. This not only means that an amino acid can change completely but also that stop codons can be added or ignored, while different codon availability can also affect the translation. Even when happening in intronic regions, editing can have a great impact, as cryptic splice sites can arise, or modulate the specificity of microRNA (miRNA) targets [17].

Furthermore, A-to-I editing has been described in a myriad of processes. A prominent example is the regulation of innate immunity in humans, which modulates the antiviral response. ADAR can edit the viral double-stranded RNA (dsRNA), thus inactivating the virus [18, 19], but this process can also dampen the interferon response (due to mismatches in the dsRNA sequence), turning ADAR into a pro-viral agent. In mice, ADAR null embryos die before birth due to stress-induced apoptosis, while ADARB1 null embryos will die young due to seizure-related complications. Alterations in the levels of editing have also been found in various diseases, such as Prader–Willi’s syndrome [20] or Alzheimer’s disease [21]. Editing in transcripts, such as *GLI1* [22], *AZIN1* [23], or *ARGHAP26* [24] (in this last case, the editing happening on a target of miRNAs) has been shown to be relevant in some cancers. The most prominently studied cases of A-to-I editing are those that affect the brain and nervous system of mammals and other vertebrates. Specifically, there are editing targets in key mediators of the synaptic transmission of neuronal signals, like the *GluA2*, *GluA3*, and *GluA4* subunits of the AMPA Glutathione receptor [25], the *GluK1* and *GluK2* kainate-glutamate receptor [26–28], or the *Nova1* splicing factor [29]. The editing in *Nova1* is a particularly noteworthy case. Nova1 is a splicing factor that regulates more than 700 splicing events, including splicing in important synaptic proteins. A specific nucleotide is a target of a conserved editing event that creates a serine-to-glycine substitution, which significantly increases the stability of the Nova1 protein. This editing event is dynamically regulated during brain development. The comparison of the editing levels in different regions of the brain shows differences in editing regulation: in *Mus musculus* there are significant regional differences in editing level, while in *Gallus gallus* all the regions have editing levels close to 100% [29], suggesting that Nova-editing could have been involved in the evolution of particular regions of the mammalian brain.

Being a process as versatile and crucial as it is [18, 30–33], whether RNA editing has shaped evolution is of great interest. However, even with the several attempts made in recent years to shed light on the evolution of this process, how (or if) RNA editing has shaped evolution is yet to be discovered [10, 12–14, 29, 34, 35]. This is mostly due to the difficulty of predicting *de novo* RNA-editing events. Little is known about the ADAR target selection mechanism besides having to be in a dsRNA region of at least 20 bp in extent [36]. It seems that secondary structure may have a great role in impeding or facilitating the action of ADAR proteins. This is more evident when looking at the high levels of editing of the adenosines in perfect dsRNA molecules in vitro [36]. These perfect dsRNA molecules have a very straightforward secondary structure, which would allow ADAR to edit every single adenosine. Some studies also suggest that a complementary sequence residing in an intron that could generate a double strand in the neighboring area of the editing site could also be necessary during editing [37]. As the target sequences or structures harboring an adenosine that has the potential to be edited are not yet clear enough, we must rely on empirical evidence for any kind of evolutionary analysis of RNA editing. This evidence comes in the form of variant calling using same-individual genomic and transcriptomic data in order to avoid polymorphisms [38], or in the form of amplification-free techniques, such as nanopore sequencing, which can identify inosines natively [39]. Even with this empirical data and checking the conservation of the primary sequence between distinct clades, however, we cannot fully ensure that the mechanism is fully conserved independently from its targets.

Here, we present a new approach to assess the functional conservation of the targeting mechanism independently of the conservation of editing sites using two machine learning algorithms, random forest (RF) and bidirectional long short-term memory (biLSTM) neural networks with an attention layer. RF is an ensemble method that allows building a classifier based on expert descriptors and, therefore, has high interpretability. On the contrary, biLSTM networks facilitate a direct approximation from sequence windows, although interpretation may not be immediate. Using available RNA-editing databases and variant calling from same-individual data from different species (*Homo sapiens*, *M. musculus*, and *Trachurus trachurus)*, we trained an algorithm to predict RNA-editing events using secondary structure and primary sequence data in a species. With this, we predicted the events from different species to assess if the target selection mechanism is conserved between the two species, or whether, although sharing a similar active domain, the ADAR mechanism changed between those species. This novel method permits approaching the, until now elusive, understanding of the editing mechanisms through evolution.

## Materials and Methods

A more detailed section can be found in Extended Methods.

### Origin of the RNA-editing and genomic data

We obtained the human and mouse RNA data from REDIportal [40] (see Supplementary Table 1 for a distribution of the human RNA-editing events according to regions), as well as the RefSeq gene notation and the standard genome assemblies (hg38 for humans and mm10 for mice). We also obtained an older version of the human REDIportal database from the authors. For mackerel, we used the DNA-seq and RNA-seq data from the Darwin Tree of Life [41], which is from the same specimen. We also used the genome assembly and gene annotation from the Darwin Tree of Life. We aligned the DNA reads using Magicblast (v1.6.0) [42] and the RNA reads using bowtie2 (v2.4.2) [43] and then used the SAMtools (v1.15.1) [44] and bcftools (v1,11) [44] libraries to obtain separately the DNA and RNA SNVs in vcf format. Then we filtered the A-to-G variants that appeared only in the RNA SNVs, filtering out the polymorphisms from the DNA SNVs. We also set a minimum depth of 10. This resulted in our accepted mackerel RNA-editing positions. A more detailed process can be found in Extended Methods.

**Table 1:** Accuracies of already existing machine learning methods found in the bibliography

Tool	Species	Year	Algorithm	Features	SeqLen	N	Acc	Sn	Sp
PAI	*D. melanogaster*	2016	SVM [54]	Handcrafted	51	244	0.7951	0.8560	0.7311
iRNA-A	*H. sapiens*	2017	SVM [55]	Handcrafted	51	6000	0.9071	0.8619	0.9523
PAI-SAE	*D. melanogaster*	2018	SVM + SAE [56]	Handcrafted+Learned	51	244	0.8197	0.8720	0.7647
iMRM	*H. sapiens*	2020	XGBoost [57]	Handcrafted	51	6000	0.9157	0.8733	0.9580
ATTIC	*H. sapiens*	2023	Ensemble learning [58]	Handcrafted	51	6000	0.9173	0.8860	0.9487
Our	*H. sapiens*	2024	Random Forest	Handcrafted	51	6620	0.767	0.7916	0.7433
Our	*H. sapiens*	2024	biLSTM+Attention	Learned	101	46,130	0.948	0.9777	0.9183

SeqLen, Sequence length analyzed; N, Number of sequences used for training; Acc, Accuracy achieved; Sn, Sensitivity achieved; Sp, Specificity achieved.

### General pipeline for constructing the random forest and neural networks datasets

We used mostly our own programs to get the datasets for both the RF and neural network approach. We extracted the pre-mRNA sequences of both coding and non-coding genes that had editing events in them, discarding those annotated sequences that are statistical outliers in length and those sequences that have more than 20% unknown nucleotides (Ns). Note that any RNA-editing events that fall outside of the selected sequences are ignored. The negative datasets for both RF and NN are randomly selected adenosines from the selected sequences that are not annotated as edited. We then predicted the secondary structure using linearfold [45]. We annotated the information about the secondary structure in two different ways: for RF, we have, for each adenosine in a dataset, a series of descriptors that give information about the features of the secondary structure, both close to the particular adenosine and for the whole molecule. For the neural networks approach, we have an input two-channel sequence, one channel with the pre-mRNA sequence and the other with the type of secondary structure feature each nucleotide is in. Once the genes file is available, with the three channels: nucleotide sequence (SEQ), linearfold secondary structure prediction (in Dot-Parenthesis format, PAIRS), and our Secondary Structure feature annotation (STRUCTURES) plus the fourth EDIT annotation channel, the first step is to go through all the genes and select each adenosine as the center of a cut window. Then we will obtain a file with a list of sequences, each centered on an adenosine, with the selected channels (in our case, we only need SEQ and STRUCTURES), where we will also add the name of the gene, the position number of the adenosine into the gene, and the editing flag (0 if not edited, 1 if edited).

In the wild, the number of unedited adenosines is much higher than the number of edited ones (98% versus 2%), and since the number of samples is very high for both classes, we have considered that the optimal solution to deal with the imbalance consists of balancing the samples for training, always choosing as many edited as unedited samples. To do this, we always select all the edited samples, and then, an equal number of unedited samples are randomly selected from the total. Finally, once we have the balanced dataset. After obtaining the balanced datasets with 50% edited adenosines and 50% unedited adenosines, we performed a random partition of the data into three parts: 70% for training, 15% for validation during training, and 15% for the final test. After partition, the three sets continue to be well balanced in positive and negative samples.

The final step for the NN data is to encode it for feeding to the bi-LSTM with attention layer model. The coding is done in two stages. We first encode each pair (nucleotide, Secondary Structure) with an integer, and then, in a second stage, we encode each integer with a One-hot code. The particularity of our approach is that to avoid storing very large files with On-hot codes, we perform this second encoding stage in RAM at runtime.

For RF, we use R 4.2.1, while for the neural networks approach, we used a biLSTM with an attention layer neural networks model implemented in keras. A more detailed process can be found in Extended Methods.

### RF

RF is a supervised ensemble algorithm based on decision trees. Each decision tree is built using a random sample of the original data (bootstrap) and a feature randomness selection; in this way, a forest of uncorrelated trees is created that will serve to make a prediction by committee with better performance than if it were an individual. Furthermore, it is possible to determine which features are most relevant to build the predictor, which makes this algorithm easy to interpret. The R package randomForest has been used as the implementation.

Before running the RF, the presence of missing values and the degree of variability of the descriptors must be checked.

The descriptors used for the RF datasets may have missing values for some variables. XClosest descriptors may have missing data when the sequence in some type of structure is less than five occurrences. In this case, the sequence is removed. Another case is when local descriptors, such as localAverageXXXSize, do not have any occurrences XXXSize, so it is not possible to compute the average. If the number of missing values is >10%, then the descriptor is removed, and otherwise, the sequence is removed.

In addition, variables that have very little variability are eliminated since they do not provide relevant information and simplify the model.

The selection of the two hyperparameters of the RF algorithm, the number of trees and the number of variables, was carried out individually for each window size of 50, 200, 500, and 1000 nucleotides and organism. In all cases, it was tuned to a maximum number of 1000 trees and a possible number of variables: 2, 4, 5, 7, 9, 13, 17, 19, 25, 30, 40, 50, 60, 90, 110, 120, all variables. The out-of-bag (OOB) score was used as a performance measure for the selection of hyperparameter values. The Gini index was used as a measure of purity to create of trees.

### biLTSM

We have used a bidirectional LSTM, which processes sequences in the two possible directions, along with an additional self-attention layer. An LSTM network, or long short-term memory [46], is a type of recurrent neural network based on a special type of recurrent unit that solves the vanishing gradient problem present in older models. The LSTM network is capable of learning relationships, both between nearby points and between points far away from the sequence. Pre-mRNA sequences, due to their secondary structure, can present these types of spatial relationships, even between distant nucleotides in the primary sequence.

Bidirectional networks usually offer better performance than unidirectional LSTMs and also treat the tokens in a sequence in a symmetrical way.

We have also added a layer of self-attention, with the aim of trying to improve performance. The attention layer is capable of assigning different weights to different positions in each input sequence, seeking to give more relevance to the positions that are most decisive when classifying the sequence.

To develop the recurrent deep learning models, we have used the LSTM implementation made by tensorflow.keras, using the layers.Bidirectional and layers.LSTM classes. A more detailed process can be found in Extended Methods.

### Software and hardware used

To carry out this work, the following programming environments and libraries were used:

(1) Python3.
(2) Spyder IDE for Python Development.
(3) Tensorflow 2.7.0 [47] y Keras 2.7.0 [48].
(4) Conda 4.11.0 and Anaconda Navigator 2.1.2 [49].
(5) JupyterLab 3.2.1. [50].
(6) Google Colab Pro. [51].
(7) The CUDA release 11.6, and cuDNN 8.3 libraries [52, 53].

We have used the following computers.

(1) Personal computer with GPU: We have used a laptop with Intel i7 processor, 16 GB RAM and 1 TB SSD disk. In addition, it has nVidia GeForce MX450 2 GB GPU.
(2) Subscription to the Google Colab Pro service, which allows you to use a virtual machine with 26 GB of RAM and a Tesla T4 GPU card with 15 GB of RAM.
(3) AMD opteron server, Processor 6386 SE, with 500 GB of RAM, 64 processors, and NVIDIA TU104GL graphics card [Quadro RTX 4000]. It was used for processing genome-wide data.

## Results

### RF and biLSTM algorithms in RNA-editing prediction

**Figure 1:**
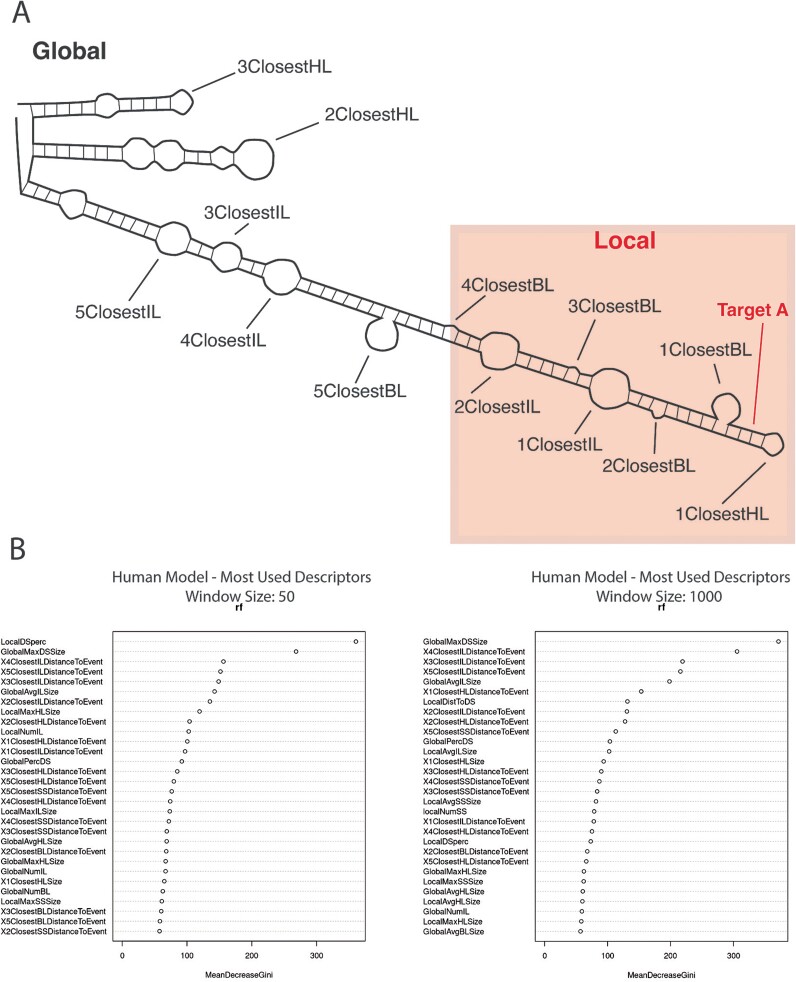
RF global and local results. (A) Schematic representation of an RNA molecule with some of the structures used as descriptors. The local window (square) provides the data for the local descriptors, while the global descriptors use the whole molecule. Target adenosine tagged in bold. The Xclosest descriptors refer to the Xth feature of that type closest to the target adenosine, independently of the local window (B) list of the most used descriptors in the RF analysis for the 50 and 1000 nt local windows using human data. See Supplementary Methods Table 1 for the complete descriptor dataset and their definition.

The use of an RF approach (see Methods) gave us the opportunity to explore the descriptors that are most commonly used to determine the potentiality of an RNA sequence to be edited. We ran four analyses with the RF algorithm, using a local window (Fig. 1A) of 50, 200, 500, and 1000 nucleotides. All window sizes performed similarly well, reaching an accuracy above 75% in the case of *H. sapiens*, around 74% in the case of *M. musculus*, and below 65% in *T. trachurus* (Supplementary Fig. 1). Nevertheless, there are some changes in the traditional descriptors depending on the window size used, especially between 50 and 1000 nt windows. Notably, the ‘global double strand maximum size’ descriptor is highly used in both cases (Fig. 1B).

**Figure 2:**
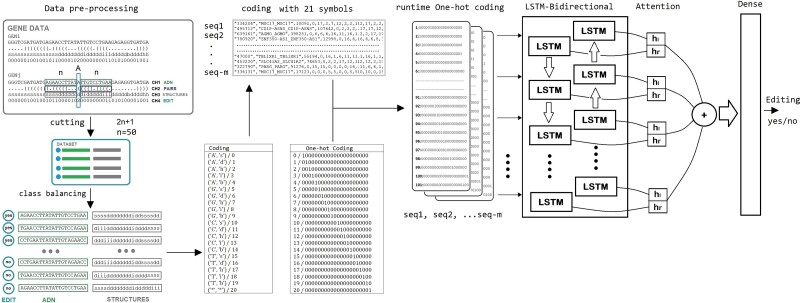
Diagram of the biLSTM data flow. From the raw data to the editability decision output. See Supplementary Methods.

**Figure 3:**
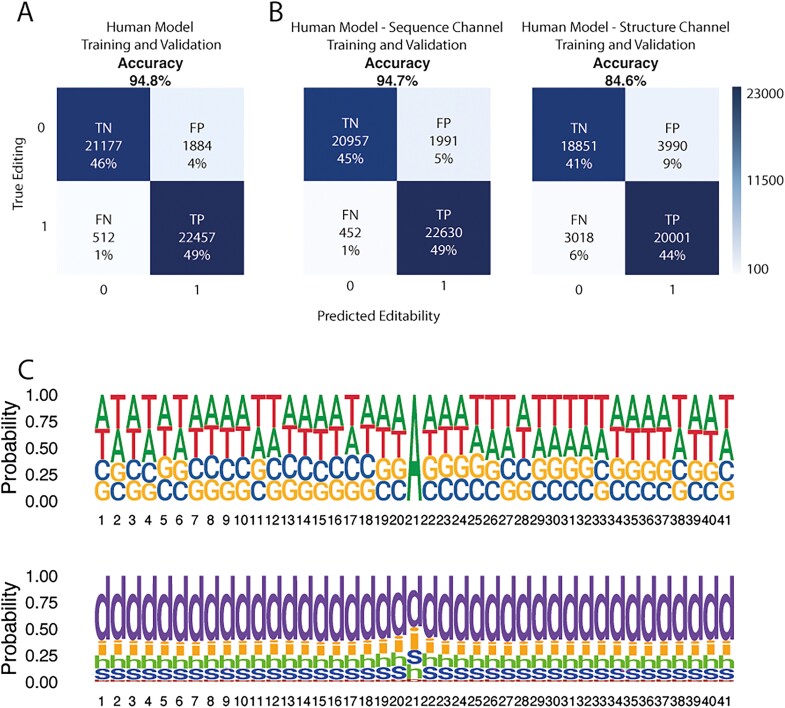
Sequence and structure channels in DL using human data. Confusion matrices for DL analysis of sequence and structure channels from human dataset combined (A) or as single-channel (B). True negative (TN), true positive (TP), false negative (FN) and false positive (FP) percentages have been rounded. (C) True positive logos for a 20 + 1 + 20 window for sequence and structure. Nucleotide 21 is the editable adenine.

On the other hand, we used a biLSTM with an attention layer (see Methods; Fig. 2) with two channels, one for the pre-mRNA sequence and another one for the predicted secondary structure. Using a sliding window of 50 + 1 + 50 nucleotides, we obtained an accuracy of almost 95% using balanced datasets (Fig. 3A). We also trained the model again using each of the two channels separately to see how they affected the ability to predict. This way, the accuracy obtained by the trained model changes when just the secondary structure channel is used (84.6%), but when using just the sequence channel, it remains similar to using both channels (94.7%) (Fig. 3B). If we explore the similarities in sequence and structure of the positive cases, we cannot see any distinguishable pattern (Fig. 3C).

### Benchmarking the algorithms with previous RNA-editing prediction attempts based on machine learning

With the human dataset predictions accuracy, we can now assess how our algorithms perform versus already existing data of machine learning predictions obtained from the bibliography [54–58]. As we can see (Table 1), although our RF algorithm does not rank near other available methods, our biLSTM algorithm is the best-performing one as well as the only one using the full extent of the REDIportal database.

### Predicting a dataset using editing proportions as in a case of *de novo* prediction of RNA-editing events

**Figure 4:**
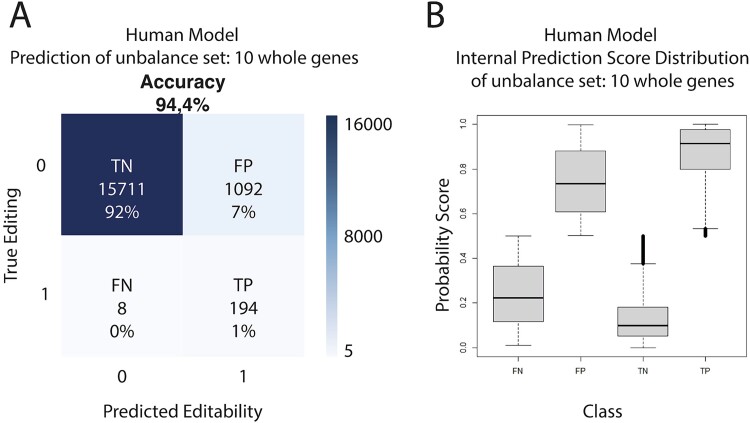
Prediction of an unbalanced dataset using DL model. (A) Confusion matrix for the prediction of editability in 10 whole human genes using DL model. True negative (TN), true positive (TP), false negative (FN) and false positive (FP) percentages have been rounded. (B) Boxplot of the internal prediction of editability score distribution for 10 whole human genes for true negative (TN), true positive (TP), false negative (FN), and false positive (FP).

Changing the balanced dataset to a dataset more akin to what we can find in a real-case scenario, gives us clues on how our prediction algorithm would perform when used for predicting new RNA-editing events. Using the full sequences of 10 random genes (as well as 20 and 30, and 100 rounds of 10 genes, see Supplementary Fig. 2), we ensure a proper data set with editing frequencies similar to the ones present in nature to benchmark our trained model. Interestingly, although the accuracy when predicting is just below 95%, the highly unbalanced nature of the dataset results in the number of false positives greatly surpassing the number of true positives (Fig. 4A). If we explore the internal score distribution, there is a slight difference between true and false positives and true and false negatives (Fig. 4B).

### biLSTM training and predictions on non-human data

**Figure 5:**
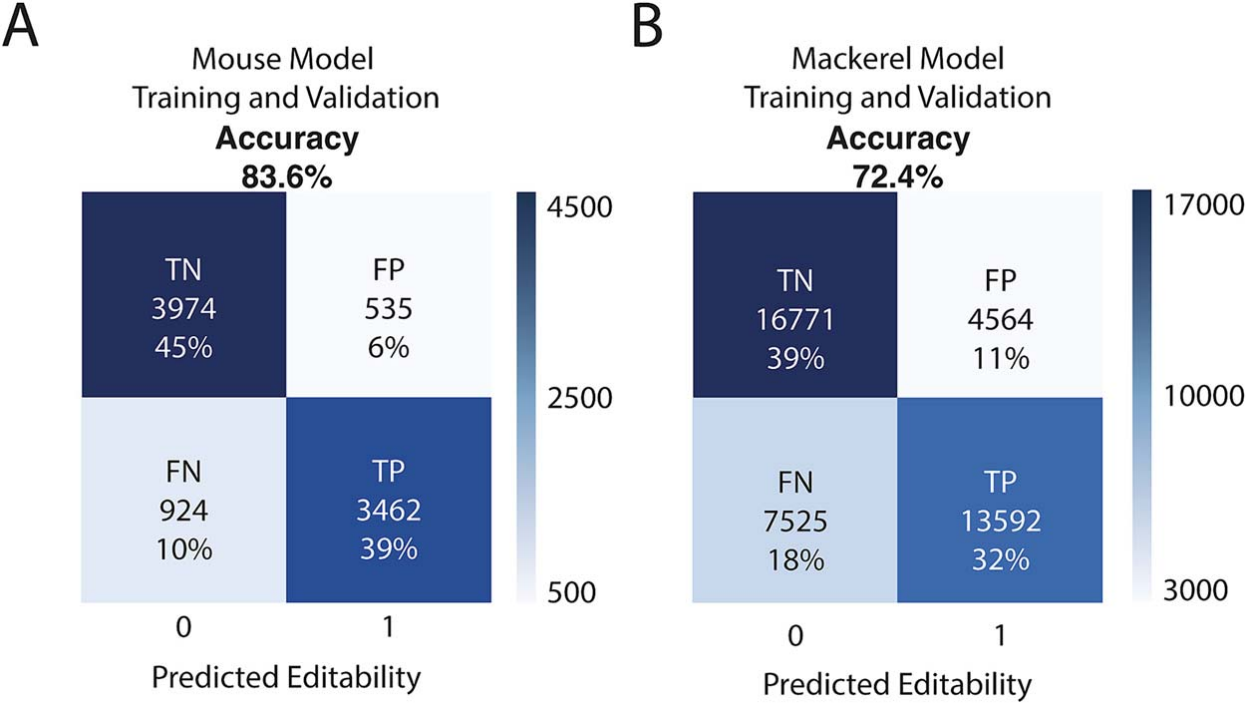
Mouse and mackerel DL analysis. Confusion matrices for models generated using mouse data (A) or mackerel data (B). True negative (TN), true positive (TP), false negative (FN) and false positive (FP) percentages have been rounded.

To further understand the RNA-editing process, we trained the model using two additional datasets, one from a mammal (*M. musculus*) and another from a teleost (*T. trachurus*). The mouse dataset came from the same database as the human dataset, albeit with fewer annotated RNA-editing events, while the mackerel dataset was obtained from the same individual RNA and DNA, thus being a narrow snapshot of the editome at the moment of collection. In both cases, the accuracy is lower than that obtained using human data, with a slight bias toward declaring an adenosine as non-editable (Fig. 5). This is especially true when using the mackerel dataset, with a 73.4% accuracy and almost 18% of RNA-editing events being flagged as non-editable adenosines (Fig. 5B).

### Cross-testing as a tool to infer mechanism conservation *in silico*

**Figure 6:**
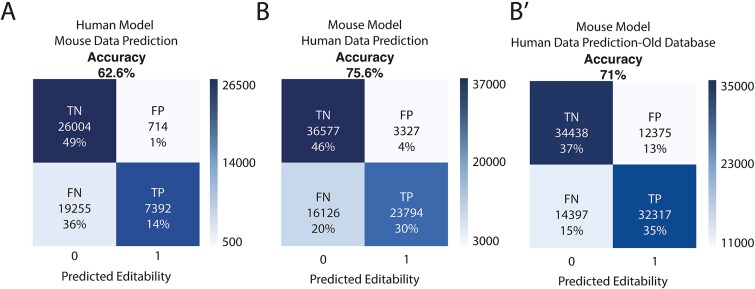
Differences of mouse model predicting in human new and old databases. Confusion matrices for models generated using human data predicting on mouse data (A) and using mouse data predicting on human data (B) and predicting on an old version of the used human database (B′). True negative (TN), true positive (TP), false negative (FN), and false positive (FP) percentages have been rounded.

With the data available, we explored how well an algorithm trained with the data of one species performs in predicting the data of the other species (Supplementary Fig. 3, Table 2). The cross-testing shows that, when trained on mammal datasets, each model can predict the other mammal’s dataset with better accuracy than the baseline of a blind random prediction (50%). When predicting *T. trachurus* data, the algorithms trained with human and mouse data achieved only 50% and 51%, respectively. Similarly, the algorithm trained with *T. trachurus* data achieved an accuracy of 52% predicting on the human dataset and 48% on the mouse dataset, with similar results obtained using the algorithm trained with human data predicting on an octopus dataset (Supplementary Fig. 4). Interestingly, although the algorithm trained on human data predicts better on humans than the algorithm trained on mouse data when predicting on mouse data (95% versus 84%), the mouse-trained algorithm predicts better on human data (76%) than the other way around (63%). Analyzing the distribution of the predictions, there is a clear tendency toward a negative prediction on the human-trained algorithm predicting mouse editable adenosines (Fig. 6A).

**Table 2:** Cross-testing accuracies of each possible pair of the three species analyzed

		Prediction		
		*Homo sapiens*	*Mus musculus*	*Trachurus trachurus*
Training	*H. sapiens*	95%	63%	50%
	*M. musculus*	76%	84%	51%
	*T. trachurus*	52%	48%	72%

## Discussion

### Using machine learning to predict RNA-editing

#### Random forest

Our data shows how a machine-learning approach is able to learn the RNA-editing signal. Although the RF approach is not as accurate as the biLSTM algorithm, it is still well above the threshold expected by a random prediction (50%, as it is a prediction between two equiprobable classes) (Fig. 1). This may be due to the fact that the descriptors used in the RF are not the most suitable for the task. Albeit they were curated by us, taking into account all the previous work on secondary structure and RNA editing [36, 59–62], some unknown descriptors may be missing. Even so, across all the different RF models, there is consistency among the most frequently used descriptors: the size of the largest double-strand fragment in the whole molecule (GlobalMaxDSSize) and the distance to the target adenosine of the fourth and fifth closest inner loops (X4ClosestILDistanceToEvent and X5ClosestILDistanceToEvent) (Fig. 1B, Supplementary Fig. 1). The GlobalMaxDSSize descriptor may be relevant for discriminating along the decision tree, as it is a value describing the whole RNA molecule. Any RNA molecule will have either a mixture of editable and non-editable adenosines or all non-editable adenosines. Thus, the global parameters may play a role in discriminating between these two groups [63]. The relevance of the fourth and fifth closest inner loops is a bit puzzling, as it is counterintuitive that distant features are more relevant than closer ones. This could again be an early discriminating descriptor between the two aforementioned groups. The most relevant local descriptor can be found when the local window is set to 50 and 200 nucleotides (Supplementary Fig. 1A and B). Here, the local percentage of double-strand (localDSperc) is clearly the most used descriptor, which may mean that, with smaller local windows, the percentage of double-stranded nucleotides around the adenosine gains importance to assess the editability. Remarkably, changing the local window affects the relevance of some of the descriptors, while achieving very similar accuracies in all cases (Supplementary Fig. 1). This could mean that, with our curated descriptors, there are several ways to predict RNA editing. In the end, we see that other than the first two or three descriptors for each model, the frequency of the other descriptors remains similar, which supports the idea of a very complex decision-making process using a high number of different input variables. Although promising, our RF algorithm falls short of achieving the accuracies observed using other machine-learning methods for RNA-editing prediction (Table 1). This could be due either to the RF algorithm used or, most probably, to the curated descriptors selected being based solely on secondary structure information obtained from LinearFold.

#### biLSTM algorithm

Looking closely at the biLSTM predictions, we can see that the accuracy using both (sequence and structure) channels is almost the same as the accuracy using just the sequence channel (Fig. 3B). But even with just the structure channel, accuracy is still well over 80%. These biLSTM input single-channel experiments allow us to infer that the secondary structure is a key element for discriminating between editing and non-editing, as suggested in previous works [36]. However, the lower accuracy when predicting with the structure channel may mean that the biLSTM algorithm is better at predicting secondary structures from the 101-nucleotide window of the sequence channel than the specialized software [45] from the complete molecule. Another option could be that using the structure channel narrows all the possible structures to one, as this channel eliminates the sequence information [45, 64]. Meanwhile, using the sequence channel would allow all the possible secondary structures to be predicted. In addition, the apparent lack of enrichment or motive seen in the separate logos for sequence and structure (Fig. 3C) must be due to the existence of multiple highly different signals that allow ADAR to access the editable adenosine, which could imply that not all the edited adenosines have a 3′ enrichment of guanosines. Although this implication seems to conflict with the results obtained in other works [35], it could be simply the use of a more diverse dataset, or the fact that in this study, we did not differentiate between ADAR editing and ADARB1 editing. Remarkably, we can see how, when compared to the existing methods (Table 1), our biLSTM algorithm performs the best in terms of accuracy using human data, even taking into account the huge differences in the training datasets, with our dataset being the whole A-to-I editing events REDIportal database.

With the data presented here, not only can we consolidate the role of the secondary structure in the RNA-editing target-selection mechanism, but we can also narrow the spatial window of the mechanism down to ±50 nucleotides from the edited adenosine [35, 37]. This is certainly true in the two species where we have successfully learned to predict with >80% accuracy, *H. sapiens* and *M. musculus*. Regarding the *de novo* prediction capabilities of both algorithms, neither is accurate enough to compensate for the huge disproportion of edited versus non-edited adenosines. One possible way of discriminating between true and false positives could be to consider their prediction score, hindering the sensitivity of the prediction in the process. While some of the false positives detected in the unbalanced dataset could indeed be non-described RNA-editing events, it remains difficult to differentiate them from the actual (and quite more frequent) false positives. But if we compare our results from human data with other analyses using experimental RNA-editing evidence from 10 human transcripts obtained from nanopores [39], we see similar levels of edited sites. In addition, Chen et al. reported only a difference of eight edited sites from the REDIportal database.

### Differences in accuracy between human and non-human data

Although *M. musculus* and *T. trachurus* prediction accuracies are significantly above random chance, they are 10% and 20%, respectively, below the accuracy obtained in *H. sapiens*. These differences could be explained by the different characteristics of the datasets, e.g. the different sizes. However, the results obtained by adjusting the training datasets to the same number of events available for *M. musculus* yielded very similar results for *H. sapiens* (Supplementary Figure 5). This means that between those two species, the different accuracies do not arise from the number of events but from the kind of events available in each dataset. In this light, we decided to train the model with an older version from the human database as, akin to the mouse dataset, it will have a more generic set of RNA-editing events than its newer counterpart (Supplementary Fig. 6) [65, 40]. The algorithm trained with the older version of the database, nonetheless, performed similarly to the newer one, with a slight decrease in accuracy, meaning that even with the same number of entries, the more uncommon editing examples available, the more accurate the predictions become. As for *T. trachurus*, we found a severe decrease in accuracy when adjusting its dataset size to the *M. musculus* dataset. This could be caused by the lower quality of the dataset, as its origin is a single RNAseq experiment (coupled with same-individual genomic data) from the Darwin Tree of Life project [41]. Nonetheless, the possibility of some of the false positives being non-described edited sites was also considered. However, due to the scarceness of editable sites when compared with non-editable sites, and the samples being balanced on editable sites, it should not impact the overall accuracy of the analysis.

### Cross-testing and mechanism conservation

One of the most promising applications derived from the machine-learning approach studied here is the inference of functional conservation completely *in silico*. With this in mind, we used cross-testing: training with datasets from one species and testing on datasets from other species. If the mechanism is fully conserved between two species (that is, the patterns that ADAR recognizes are the same) with a similar completeness database, the accuracy between their cross-testings should be similar. Here, we see how this happens between the cross-testings from the old and the new human databases [65, 40] (Supplementary Fig. 6). In the case of the cross-testings between *H. sapiens* and *M. musculus*, we show how, although similar, the mouse-trained algorithm performs better on the human dataset than the other way around. The reason for this may be a minor functional difference coupled with the aforementioned lower completeness of the database from *M. musculus*. From the bias toward false negatives present in the human-trained algorithm, it may be inferred that mouse-specific structures are being misclassified as non-editable. This would explain the higher balance between false negatives and false positives when predicting the human dataset with a mouse-trained algorithm, as well as the already mentioned bias and the different accuracies (Fig. 6). These differences are more obvious when cross-testing the old human database with *M. musculus* (Fig. 6B′) and are also observed in the RF cross-testing tests, where the mouse-trained algorithm predicting on human data outperforms the human-trained algorithm predicting on mouse data (Supplementary Fig. 7).

For *T. trachurus* cross-testing, in all cases, the accuracy is around 50%, which is expected for a random prediction [66]. While the accuracy of the biLSTM algorithm trained in *T. trachurus* was not as high as the one from human or mouse, we did not expect such low performance in the cross-testings. The main reason for the inability to predict in *T. trachurus* when training the algorithm in human or mouse (or the other way around) may well lie in the differences in homeostatic temperature affecting the secondary structure of the RNA molecules [67], with similar results obtained in cross-testing using human data to predict octopus editable sites (Supplementary Fig. 4). If we analyze the single-channel biLSTM results from *T. trachurus*, we can see how we fail to predict above random chance when using just the secondary-structure channel (Supplementary Fig.8). This could mean that the secondary structure prediction software used [45] is not working as intended in the case of the cold-blooded mackerel, with the biLSTM algorithm completely relying on the sequence channel.

Our results demonstrate the power of machine learning approaches to predict RNA editing events. However, despite the extremely high accuracy reached, we are not yet able to use these algorithms to predict de novo editing events reliably due to the unbalanced nature of edited versus non-edited adenosines. Nonetheless, thanks to our new cross-testing approach, we can further understand the differences in RNA editing between different species, and how these differences could have shaped evolution. This opens the door to investigate whether some species have a fast-evolving RNA-editing machinery, or if the absence of one of the ADARs can reshape the RNA-editome.

**Key Points**

- Due to the imbalanced nature of RNA-editability versus non-editability *de novo* prediction needs higher accuracy than 95%.
- Cross-testing allows us to infer the conservation of the RNA-editing mechanism between species.
- While the algorithms perform well with secondary structure data, the information within the sequence is enough for the biLSTM with attention layer algorithm to reach extremely high levels of accuracy.

## Supplementary Material

Supp_Figures_and_Tables_REVIEWED_3_BiB_bbae332

Supplementary_Methods_Review_3_BiB_bbae332

## Data Availability

The authors declare that all data supporting the findings of this study are available within the paper and its supplementary information files. The software used can be found in: https://github.com/cherrera1990/RNA-editing-pred.

## Author Biographies

**Michał Zawisza-Álvarez** is currently a PhD student at the University of Barcelona. His interest are evo-devo and machine learning.

**Jesús Peñuela-Melero** worked as a Master’s student on this project and specializes on deep learning and LSTM neural networks.

**Esteban Vegas** is a senior lecturer in the University of Barcelona. His research interests are machine learning and deep learning applied to biomedical data.

**Ferran Reverter** is a lecturer in the University of Barcelona. His research interests are machine learning and deep learning applied to biomedical data.

**Jordi Garcia-Fernàndez** is a professor and group leader in the University of Barcelona with a long and distinguish career in the field of evo-devo. His research interests are bioinformatics, evo-devo and WGD events.

**Carlos Herrera-Úbeda** is currently a postdoctoral fellow in the University of Barcelona. His interests are bioinformatics, evo-devo and evolution of RNA-related mechanisms.
